# Stakeholder involvement in a Cochrane review of physical rehabilitation after stroke: Description and reflections

**DOI:** 10.1002/cesm.12032

**Published:** 2023-12-01

**Authors:** Julie Brown, Gill Baer, Sheila Cameron, Karl Jackson, Carrol Lamouline, Richard Morley, Diane Ormsby, Anneliese Synnot, Alex Todhunter‐Brown

**Affiliations:** ^1^ Physiotherapist Glasgow UK; ^2^ Division of Dietetics, Nutrition and Biological Sciences, Physiotherapy, Podiatry and Radiography Queen Margaret University Edinburgh UK; ^3^ Chair, NMAHP Research Unit Research Partnership Group Glasgow Caledonian University Glasgow UK; ^4^ Consultant Stroke Therapist, Bangor Hospital Betsi Cadwaladr University Health Board Bangor UK; ^5^ Lay Contributor London UK; ^6^ Consumer Engagement Officer Cochrane UK; ^7^ Balance Neuro Physiotherapy Fareham and Gosport UK; ^8^ School of Public Health and Preventive Medicine Monash University Melbourne Australia; ^9^ Nursing, Midwifery and Allied Health Professions (NMAHP) Research Unit Glasgow Caledonian University Glasgow UK

**Keywords:** coproduction, patient and public involvement, reflection, stakeholder involvement, stroke, systematic review

## Abstract

**Introduction:**

It is good practice to involve stakeholders in systematic reviews, but it is not clear how best to involve them.

**Aim:**

To describe and reflect on the stakeholder involvement within an update of a Cochrane review of physical rehabilitation after stroke.

**Methods:**

A stakeholder group, comprising 15 stroke survivors, carers, and physiotherapists from across the United Kingdom, were recruited and contributed throughout the process of the review. A framework was used to describe when and how stakeholders were involved. Stakeholders provided feedback on their involvement after meetings. An amended version of a validated patient engagement tool was used to collect reflections on the stakeholder involvement process.

**Results:**

Five stakeholder meetings were held throughout the review process, supplemented by additional communication. Several changes were made to the review structure, analyses, and wording as a direct result of the stakeholder involvement. Stakeholders and researchers agreed that stakeholders' contributions were taken seriously and influenced the review. Stakeholders felt that they were given the chance to share their views and that information was shared well before, during, and after each meeting to help them to contribute knowledgeably in the process. Stakeholder reflections highlighted a number of key lessons relating to stakeholder involvement, including process of reflection and feedback, use of remote/virtual meetings, need for adequate time and funding, tensions experienced by clinicians, and recruitment considerations.

**Conclusions:**

We describe and reflect on stakeholder involvement in a systematic review and explores practical ways to support meaningful engagement during systematic review production. Our experience supports the view that coproducing reviews with stakeholders can make systematic reviews more relevant and meaningful. Our approach and experiences can be used to inform future review coproduction, supporting development of useful reviews that will improve clinical practice.

## 1 INTRODUCTION

Including the views and opinions of a wide range of different people in health research is increasingly seen as a fundamental good practice principle, expected—and considered important—by people who use evidence, and often mandated by funders of research [1]. The people who it is generally considered important to involve can be referred to as “stakeholders” in the research. A “stakeholder” has been defined as “an individual or group who is responsible for or affected by health‐ and healthcare‐related decisions” [2], and includes patients, carers, the public, health professionals and others, such as healthcare policymakers or managers. Stakeholder involvement in health research is different from involvement of people as participants in a study, where data are collected from people who are the subjects of the research [1, 2].

The terms used to refer to, and definitions of, “involvement” in research vary internationally [1]. The UK's National Institute of Health Research (NIHR) defines involvement as “Research being carried out ‘with’ or ‘by’ members of the public rather than ‘to,’ ‘about’ or ‘for’ them. It is an active partnership between patients, carers and members of the public with researchers that influences and shapes research” [3]. Within this paper we use the phrase “stakeholder involvement” to refer to the involvement in research of any people who have an interest in the research, in partnership with the (traditional) research team. We recognize that terminology varies and is sometimes contested, but we consider this phrase and definition to be widely used and understood. The term “coproduction” is also used within this paper; this has been defined as “working together in a ‘partnership’ to produce research” [1] and we consider this one form of stakeholder involvement, in which specific criteria are met in relation to partnership working [4].

Stakeholder involvement is widely acknowledged as beneficial across health and social care research, for example, clinical trials [5], qualitative studies [6], and guideline development [7]. There is growing recognition that it is also good practice to involve stakeholders in systematic reviews [8]. Systematic reviews, sometimes called evidence synthesis, are “…a way of combining information from multiple studies that have investigated the same thing, to come to an overall understanding of what they found” [9]. Despite initiatives such as Cochrane's ACTIVE (Authors and Consumers Together Impacting on eVidencE) project, which produced a framework to describe stakeholder involvement in a review [10] and an open access learning resource focused on methods of involvement in reviews [11], there remains uncertainty about how best to actively involve stakeholders in systematic reviews [7, 12].

Stakeholder involvement was central to an update of a Cochrane systematic review relating to physical rehabilitation after stroke, published in 2014 [13]. A group of stroke survivors, carers, and physiotherapists contributed to key decisions about the review aims and methods [14]. The methods of involving stakeholders in this Cochrane review update, which were informed by previously published strategies [15] have been reported within the ACTIVE learning resources and cited as an exemplar of coproduced research [16]. This 2014 Cochrane review is considered a top priority review, with evidence of high access (in the top three most accessed Cochrane Stroke reviews, 2014–2017 and in the top 50 accessed Cochrane reviews in 2016 and 2017) and citation within multiple national and international clinical guidelines [17, 18, 19]. Top priority reviews should be updated if they are missing evidence from new trials. A scoping search conducted in 2019 highlighted that the published version of this review may be missing hundreds of new trials, and consequently a comprehensive update was initiated (due for publication 2023). To ensure continued relevance and impact of this updated review, stakeholder involvement was again planned, this time with plans informed by the ACTIVE framework and resources. The overall goal of the stakeholder involvement in this 2023 review update was “to guide progress and ensure relevance and accessibility of the output” [20].

The aim of this paper is to describe and reflect on the stakeholder involvement within the 2023 update of this Cochrane review.

## 2 METHODS

### 2.1 Overview of methods of involving stakeholders within the Cochrane review

The prestated [20] objectives for stakeholder involvement in the 2023 update of the Cochrane review of physical rehabilitation were to:

a. Update and inform the description and categorization of physical rehabilitation following stroke and make it useful and accessible to all interested parties.
b. Ask the right questions in physical rehabilitation research, informing the structure and conduct of analyses and subgroup analyses within the Cochrane review of physical rehabilitation following stroke.
c. Consider implications for clinical practice arising from the results of the Cochrane review of physical rehabilitation following stroke.
d. Help shape plans for dissemination of the synthesized research evidence, so that it reaches—and is useful to—the right people/organisations.

These specific objectives informed the methods of stakeholder involvement, including formation of the stakeholder group, plus the timing of, and agendas for, each of the stakeholder meetings that were held during the course of the review. A recruitment process led to the formation of a stakeholder group, comprising 15 members from the United Kingdom, who contributed to a series of five preplanned meetings throughout the process of the review.

As the aim of this paper is to describe the methods of stakeholder involvement throughout the review, further details of the group recruitment, membership, and meetings are provided in the results section, while the methods section focusses on the collection and synthesis of data for this paper.

### 2.2 Methods of describing our stakeholder involvement approach

To describe the stakeholder involvement approach, we use the ACTIVE framework [10]. This framework provides a structured way of describing the methods of stakeholder involvement in an evidence synthesis. Key components include documentation of who was involved, how they were recruited, what they did, when within the review process they did it, and the level of control or influence stakeholders had over decisions relating to the review.

### 2.3 Methods to document stakeholder reflections

#### 2.3.1 Data collected after stakeholder group meetings

Feedback and reflections were collected after the first two meetings, via typed forms, over telephone or email dependent on the communication needs/preferences of the stakeholder (see Supporting Information S1: Appendix 1 for feedback/reflection form). Before, during, and after all other meetings, stakeholders were encouraged to provide informal feedback, using their preferred medium, but the formal reflection form was not used.

#### 2.3.2 Reflections on the whole project

An amended version of a validated patient engagement tool, the public engagement evaluation tool (PEET6 tool) [21] was used at the end of the project to collect reflections from all stakeholder group members. Before use, the stakeholder authors on this paper proposed, refined, and agreed some amendments to the PEET6 tool wording to make it easier to understand. Seven questions aimed at capturing stakeholder reflections were agreed on (see Table 3) and posed to all members of the stakeholder group and research team. Reflections were collected during a meeting (on recorded video call), after the meeting via email, or via an anonymous online message board (“Padlet”).

#### 2.3.3 Methods of coproducing this paper

All 15 members of the review stakeholder group were invited to coproduce this paper. Four people (SC, DO, CL, and KJ) volunteered to do this, in partnership with five members of the research team (JB, ATB, AS, RM, and GB). These four stakeholders attended a meeting at which they discussed and agreed key points for the paper and preferred ways of work together. This involved collaborative writing: one stakeholder preferred providing verbal feedback and contributions (done by calls with a research team member [JB]), while others did this in writing. One researcher (JB) coordinated writing activity and feedback, writing drafts that brought initial ideas together and leaving prompts for the subgroup to respond to, ensuring all were able to contribute in their own words. Thus, the paper developed in an iterative manner, with all authors contributing to writing.

## 3 RESULTS

### 3.1 Who was involved

Four stroke survivors, four carers, and seven physiotherapists were recruited to the stakeholder group. Stroke survivors had had their strokes between 6 and 28 years previously, and carers of stroke survivors had caring experience between 2 and 18 years previously. Three physiotherapists had more than 15 years of experience of physical rehabilitation after stroke; two had 10–15 years' experience and two had 5 years or less experience. Stakeholders were from England (*n* = 7), Scotland (*n* = 6), Wales (*n* = 1), and Ireland (*n* = 1). There were two dropouts from the stakeholder group: One stroke survivor withdrew because they found it too difficult to engage with the complex discussion; one physiotherapist withdrew due to personal reasons.

### 3.2 How stakeholders were recruited and selected

**Figure 1:**
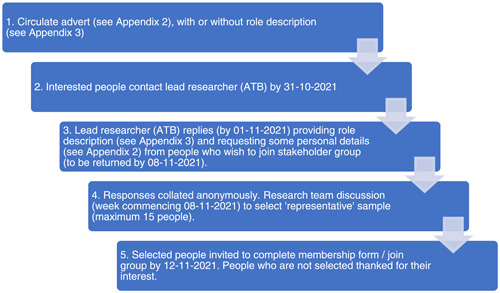
Recruitment process.

Stakeholder group members were recruited through local and national networks of the research team, including cascading the advert via social media. Figure 1 summarises the recruitment process; 72 people (52 health professionals, nine stroke survivors, nine carers, and seven others/not stated) responded to the adverts and were sent further information; 38 people (23 health professionals, eight stroke survivors, and seven carers) responded by providing the requested personal details; 15 people (seven health professionals, four stroke survivors, and four carers) were invited and agreed to join the stakeholder group.

See Supporting Information S2: Appendix 2 provides our adverts and requests for information from potential participants and Supporting Information S3: Appendix 3 provides role descriptions circulated to participants (Table 1).

**Table 1:** Glossary of terms that we use in this paper.

Term	What we mean when we use this term
Stakeholder	Within this paper, we use the phrase “stakeholder involvement” to refer to the involvement in research of any people who have an interest in the research working in partnership with the (traditional) research team. Other terms familiar to readers may be patient and public involvement (PPI) or consumer involvement (consumer being the preferred term of Cochrane when referring to patients and the public who use evidence from Cochrane reviews).
Stakeholder group	The team of stakeholders who worked on this review and attended multiple meetings and were involved. Other terms familiar to readers may be “stakeholder advisory group,” “patient and public involvement group.” Stakeholders in this group were stroke survivors, carers, and physiotherapists—the end users of research, able to use their lived experience of physical rehabilitation after stroke to inform the review.
Stakeholder paper subgroup	Members of the research team and stakeholder group who expressed an interest to volunteer to coproduce this paper.
International webinar stakeholder	Stakeholders who attended an international webinar and were given the option to feedback on the framework to describe physical rehabilitation after stroke. This was one time involvement.
Coproduction	The term “coproduction” is also used within this paper; this has been defined as “working together in a ‘partnership’ to produce research” [1], and we consider this one form of stakeholder involvement, in which specific criteria are met in relation to partnership working
“Framework to describe…”	When explaining how we organized the different parts of physical rehabilitation after stroke to categorize the interventions within the review, we used a “framework to describe“ physical rehabilitation after stroke to explain how the different parts of rehabilitation came together. Others may understand this as a “conceptual framework” but the lay term of “framework to describe” is used in this paper as a suitable plain language description.
Stroke survivors	We use the term stroke survivors throughout this paper to refer to people who have had, and survived, a stroke. This term is commonly used in stroke research.
Carer	We use the term carer to refer to a close family member or friend of a stroke survivor. Other terms include family and friends.
Systematic reviews	Systematic reviews are also sometimes called evidence synthesis. They are “…a way of combining information from multiple studies that have investigated the same thing, to come to an overall understanding of what they found” [9].

### 3.3 What stakeholders and researchers did and when in the review process they did it

**Figure 2:**
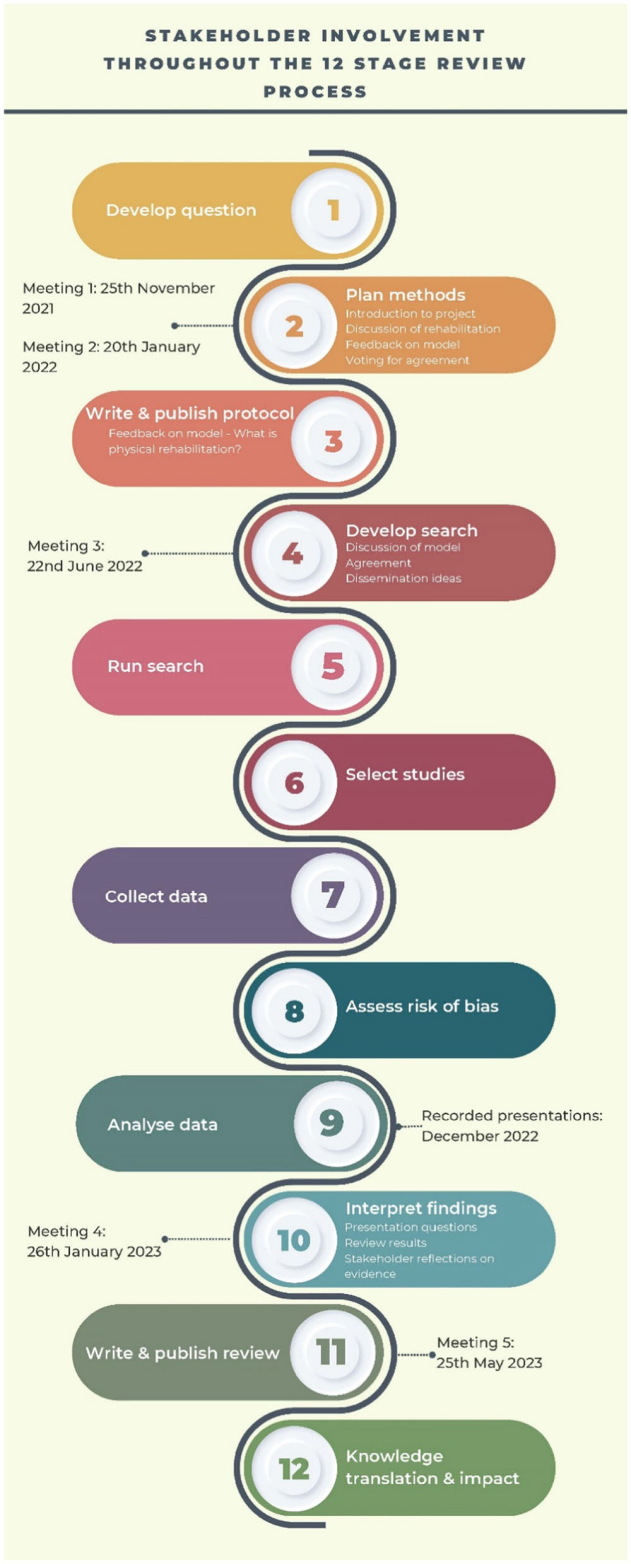
Stakeholder involvement throughout the review process.

Figure 2 illustrates when stakeholder involvement was undertaken across the review process and Table 2 provides further information about involvement and activities, including.

**Table 2:** Overview of stakeholder meetings and activities across the review stages.

Review stage (date)	Activity	Who attended	Key items discussed/key actions
Plan methods (November 25, 2021)	**Stakeholder meeting 1** Video meeting using Microsoft teams	14 stakeholders 4 researchers	At meeting: ‐ Introduction to project ‐ Discussion about meeting rules [see Supporting Information S4: Appendix 4] and ways of working, subgroup analyses, approaches to physical rehabilitation, IT issues, how to claim expenses ‐ Feedback on how the meeting ran After meeting: ‐ Email sent requesting feedback on how meeting ran [see Supporting Information S1: Appendix 1] and online vote on a Microsoft Form. ‐ Six people provided feedback
Plan methods (January 13, 2022)	**Meeting:** IT troubleshooting meeting using Microsoft teams	2 stakeholders 4 researchers	At meeting: ‐ Informal session ‐ Chance to ask Glasgow Caledonian University IT team questions about using Microsoft Teams After meeting: ‐ Troubleshooting advice document circulated to whole group
Plan methods (January 20, 2022)	**Stakeholder meeting 2** Video meeting using Microsoft teams	13 stakeholders 4 researcher	One week before meeting stakeholders received: ‐ Recorded presentations about how we could describe physical rehabilitation after stroke, and slides of presentations for meeting 2 At meeting: ‐ Recap from meeting 1 and repeat of presentations ‐ Discussions about: framework to describe physical rehabilitation after stroke, review title, definitions used in review, volunteers requested to help write definitions for additional components of physical rehabilitation, how to share files After meeting: ‐ Microsoft teams group set up to share files, meeting reflection forms sent out via email ‐ Five people provided feedback
Develop search (June 22, 2022)	**Stakeholder meeting 3** Video meeting using Microsoft teams	10 stakeholders 4 researchers	At meeting: ‐ Presentation of update on review (initially planned to share initial results but project running behind, and consequently there were no results available at this time) ‐ Discussions about terminology used for framework to describe physical rehabilitation after stroke, comparisons in review, project timelines, dissemination ideas,
Analyse data and interpret findings (December 2022)	**Recorded presentations**	Emailed to all stakeholder group to watch at preferred time	Recorded presentations on framework to describe physical rehabilitation after stroke. Included three presentations: (i) review update, (ii) forest plots training, and (iii) new coproduced framework to describe physical rehabilitation after stroke ‐ Stakeholders gave feedback on presentations requested via email or on anonymous online discussion board (Padlet).
Interpret findings (January 26, 2023)	**Stakeholder meeting 4** Video meeting using Microsoft teams	9 stakeholders 3 researchers	At meeting: ‐ Presentation of review results ‐ Discussion about results including wording of key messages and how to collect stakeholder reflections After meeting: ‐ Request for reflections via anonymous online discussion board (padlet) or via email
Interpret findings and write & publish review (May 25, 2023)	**Stakeholder meeting 5** Video meeting using Microsoft teams	7 stakeholders 5 researchers	At meeting: ‐ Presentation of recap of review and results ‐ Discussion about results including wording for dissemination and clinical implications, and key findings

There were some differences between the planned and conducted meetings. These differences included organizing additional meetings (one to trouble‐shoot IT issues, and another due to a project extension), and replacing planned meetings with recorded presentations (due to researcher illness).

### 3.4 Changes to the review as a result of the stakeholder involvement

We noted several changes to the review as a direct result of stakeholder involvement. Key changes included:

- **A new framework for describing physical rehabilitation treatment components**. Stakeholders identified that key components were missing from previous descriptions of physical rehabilitation treatments within the review. This led to coproduction of a new framework to describe physical rehabilitation after stroke, which was used to categorize evidence in the review.
- **Subgroup analyses**. Stakeholders directly informed decisions relating to some additional, and amended, subgroup analyses within the review.
- **Interpretation of results**. Stakeholders provided feedback on preliminary review results. This directly informed changes in the language used to articulate the findings. For example, information about the certainty in the evidence was clarified and key reasons for downgrading evidence were provided.
- **Discussion of findings**. Stakeholders directly informed several points within the review discussion. For example, a discussion relating to the importance of healthcare variations and differing cultures. Stakeholders also discussed and agreed wording of key implications for practice used in the conclusion section of the review.
- **Plain language summary**. Stakeholders provided written feedback on an initial draft of the plain language summary, written by the lead review author, leading to substantial changes to wording and sentence structure. Further, information was added relating to findings pertaining to the dose of physical rehabilitation, and highlighting where evidence suggests that one approach may be less effective than another.

### 3.5 Stakeholder reflections

Details of stakeholders providing feedback after meetings are shown in Table 2. Stakeholders felt that meetings were well managed, with opportunity to express views, and have an “open, honest and engaging” discussion. However, IT issues affected some members of the team and attempts at online voting had to be abandoned due to IT issues. Stakeholders considered that their input changed or influenced the review. When asked about their perceived level of control, based on the ACTIVE framework, stakeholders indicated a wide range of perceived levels (ranging from controlling to receiving).

Reflection on the coproduction process as a whole were gathered from three people via the anonymous online noticeboard (Padlet) and five via email. In addition, authors to this paper contributed further reflections. Table 3 summarises key stakeholder reflections in response to the PEET6 reflection questions, with more detail provided in Supporting Information S5: Appendix 5.

**Table 3:** Overview of stakeholder meetings and activities across the review stages.

Reflection questions posed to all stakeholders (based on public engagement evaluation tool PEET6 [21] questions)	Summary of stakeholder responses (see Supporting Information S5: Appendix 5 for further detail)
1. Do you believe that your ideas were heard during the engagement process?	Stakeholders felt their ideas were welcomed and they could see how these were taken up. Researchers agreed with this.
2. Do you believe organizers took your contributions to the engagement process seriously and that your contributions influenced final decisions on the project?	Stakeholders felt that meeting notes and recapping on ideas and actions made them aware of the impact they were having. Researchers agreed with this.
3. Did feel you were able to clearly express your viewpoints and all participants were given equal opportunity to participate in discussions?	Stakeholders mostly felt able to clearly express their viewpoints and the ‘ground rules’ helped, but the discussions were complex and the online meetings created some challenges.
4. To what extent was information made available to you either before or during the engagement process to help you participate knowledgeably in the process?	Stakeholders felt information was shared well before, during and after each meeting, although it was sometimes challenging to understand. Prerecorded presentations were considered helpful.
5. What could we have done differently to make you feel that you were valued, important, and heard?	Researchers felt they could have planned for more time to support the stakeholders. Stakeholders did not respond to this question.
6. If relevant, was your ethnicity or culture considered?	Researchers highlighted that they did not consider ethnicity or culture when recruiting stakeholders. Stakeholders did not respond to this question.
7. Do you feel there was a wide enough group of consumers from all backgrounds with good representation and diversity?	One stakeholder responded that there was a wide enough representation of diverse backgrounds with a rich mix of professionals, stroke survivors, and carers. Researchers felt there was diversity, but reflected on limitations of the recruitment approach.

## 4 DISCUSSION

A team of carers of stroke survivors, stroke rehabilitation physiotherapists, and systematic review researchers have cowritten this paper. We describe and reflect on the stakeholder involvement within an update of a Cochrane review of physical rehabilitation after stroke. We recruited stroke survivors, carers, and physiotherapists to a stakeholder group to inform decisions about the review. Stakeholders and researchers felt that stakeholders were heard, influenced the research, given equal opportunity to participate, and impacted the way that the review was conducted and reported. The involvement of stakeholders is perceived—by both the stakeholders and the researchers—to have enhanced the clinical relevance and accessibility to end users of research.

The research team and stakeholders reflected on the stakeholder involvement in the review. Key lessons learnt from this reflection include:

- “Real‐time” reflection (i.e., feedback after each meeting) enables adaptation of methods of involvement, optimizing engagement, but response rates may be low when stakeholders don't see the value in providing feedback on the “process.”
- Remote/virtual meetings are often considered positive, due to lack of travel and less time required, but plans should be in place to support stakeholders to deal with the challenges.
- Challenges in remote meetings, such as technical knowhow, access, and communication, need mitigation strategies in place to avoid, or reduce, potential feelings of exclusion. This is supported by other studies, e.g. [22]. Strategies considered helpful included:
  o Regular “check ins” with meeting participants, asking individuals for a contribution during meetings.
  o Providing information in advance of meetings (perhaps as a prerecorded presentation) to enable time for people to gather their thoughts and ideas.
  o Use of breakout rooms to enable smaller group discussions.
  o More frequent, shorter, meetings (rather than less frequent, longer meetings)
  o Ensuring that IT support is available before and during meetings, to help people overcome difficulties and enhance confidence and familiarities with online platforms.
- During funding applications, as recommended by others [23], cost in adequate time for co‐ordinating stakeholder involvement. Despite preplanning and funding, researchers felt they sometimes had insufficient time to coordinate the group in an optimal way, and stakeholder involvement could have been more impactful with additional time.
- Clinicians can experience tensions trying to balance clinical facing time and research commitments; this may relate to research culture in rehabilitation [24]. It is important the benefits of being involved in research are clearly recognized. Evidence highlighting how involvement contributes towards continuing professional development and advanced clinical practice [25, 26, 27, 28] would be beneficial.
- Recruitment should carefully consider diversity, both in terms of ethnic background and key characteristics relating to lived experience.

Use of reflection on the systematic review process overall is relatively uncommon however a recent review of stakeholders perspectives on engaging in systematic reviews identified similar challenges to those identified by our team of researchers and stakeholders [29]. Stakeholder reflections on their level of control over the review varied; this variation may indicate different levels of involvement by different individuals or different views on impact of stakeholder involvement. There remains lack of knowledge about the benefits and challenges of giving stakeholder different levels of control over a review; further work is needed to explore optimal approaches and how to record this.

Despite our best efforts to thoroughly document and reflect on the stakeholder process for this review, there were some limitations. Limited feedback was collected after the meetings, and not all PEET6 reflection questions were answered by all stakeholders. Further, contributions of stakeholders to coproduction of this paper were voluntary, as this paper had not been costed within the funding application which supported the review update. Notably, no stroke survivors volunteered to coproduce this paper; while barriers to their involvement were not explored, we speculate that this might relate to people's motivations for involvement, with stroke survivors being highly motivated to contribute to stroke‐related to research but less invested in a paper relating to methods of involvement. It could also be postulated that the research team did not make the paper writing accessible, or easy, enough for stroke survivors to engage in, and greater consideration and planning should be put into co‐producing papers in future. Consequently, the views expressed by the stakeholders who volunteered to contribute to this paper may not reflect those of the wider stakeholder group. We note that the researchers in this team had previous experience in stakeholder involvement in reviews; researchers new to involving stakeholders may experience further challenges. We recommend that the experience of the research team in relation to stakeholder involvement is considered at the review planning stage.

All stakeholders involved in this review were based in the United Kingdom. To explore whether decisions informed by this UK‐based stakeholder group were internationally relevant, during the course of this review update we held two international webinars at which the proposed new framework for describing physical rehabilitation treatment components was presented and discussed; we chose not to report on this information within this paper as it was felt it restricted the flow of the information about the involvement of the stakeholder group. This paper has not reflected on the potential limitations of having a UK‐based stakeholder group, or on the process of gaining international input. This is an important area which requires further exploration.

## 5 CONCLUSION

This paper describes and reflects on stakeholder involvement in a systematic review, and explores practical ways to support meaningful engagement during systematic review production. Our experience supports the view that coproducing reviews with stakeholders can make systematic reviews more relevant and meaningful. By describing our experiences we hope we can inform future review coproduction, supporting development of useful reviews that will improve clinical practice.

## AUTHOR CONTRIBUTIONS

**Julie Brown**: Conceptualization; data curation; methodology; project administration; resources; writing—original draft; writing—review and editing. **Gill Baer**: Funding acquisition; methodology; writing—review and editing. **Sheila Cameron**: Conceptualization; writing—original draft. **Karl Jackson**: Conceptualization; writing—original draft. **Carrol Lamouline**: Conceptualization; writing—original draft. **Richard Morley**: Conceptualization; funding acquisition; methodology; writing—review and editing. **Diane Ormsby**: Conceptualization; writing—original draft. **Anneliese Synnot**: Funding acquisition; methodology; writing—review and editing. **Alex Todhunter‐Brown**: Conceptualization; funding acquisition; methodology; project administration; resources; supervision; writing—original draft; writing—review and editing.

## CONFLICTS OF INTEREST STATEMENT

Alex Todhunter‐Brown is co‐lead of the Cochrane Heart, Stroke & Circulation Thematic Group and Cochair of the Cochrane Coproduction Methods Group; Richard Morley is Chair, International network on public involvement in health and social care research; Sheila Cameron and Carrol Lamouline both received payments (in the form of gift vouchers) for involvement in activities associated with the updating of the Cochrane review (as described in the paper), but did not receive any payments for contributions to this paper. Gill Baer, Julie Brown, Karl Jackson, Diane Ormsby and Anneliese Synnot declare no competing interests.

## PEER REVIEW

The peer review history for this article is available at https://www.webofscience.com/api/gateway/wos/peer-review/10.1002/cesm.12032.

## ETHICS STATEMENT

Ethical approval was granted by Glasgow Caledonian University Nursing Department Research Ethics Committee (HLS/NCH/21/001).

## Supporting information

Supporting information.

## Data Availability

Data sharing is not applicable to this article as no new data were created or analyzed in this study (other than those presented in this paper). Data relating to the updated Cochrane review will be available in the Cochrane Library when the review is published (currently undergoing editorial peer review).
