# Supplementary material for: Stakeholder involvement in a Cochrane review of physical rehabilitation after stroke: Description and reflections
Source: Cochrane Evid Synth Methods. 2023 Dec 1;1(10):e12032. doi: 10.1002/cesm.12032 (PMC11795892; doi:10.1002/cesm.12032)
Supplement: Supplementary file 1 — Supporting information. [file CESM-1-e12032-s001.docx]

**Supplementary information**

**Appendix 1: feedback / reflection sheet**

**SPRUCE: record of stakeholder involvement**

Date of involvement: ________________

| **I:** | tick |
| --- | --- |
| Have experience of rehabilitation after stroke |  |
| Am a healthcare professional |  |
| Other (please state) |  |

I would describe my role at this point in the project as:

| **Role** | I am: | tick |
| --- | --- | --- |
| **Leading** | Initiating the review; taking lead responsibility for carrying out and completion of review. |  |
| **Controlling** | Working in partnership with researchers, with varying degrees of control or influence over the review process. Making decisions and/or controlling one or more aspects of the review process, in collaboration with or under the guidance of the review authors. |  |
| **Influencing** | Stating, commenting, advising, ranking, voting, prioritising, reaching consensus. Providing data or information which should directly influence the review process, but without direct control over decisions or aspects of the review process. |  |
| **Contributing** | Providing views, thoughts, feedback, opinions or experiences. Providing data or information which may indirectly influence the review process. |  |
| **Receiving** | Receiving information about the systematic review, or results of the review. |  |

| Thinking of your involvement in this meeting / event: | |
| --- | --- |
| Do you think your involvement changed or influenced anything?    If so, in what way? |  |
| What was good? |  |
| What was not so good? |  |
| What would you change? |  |

Do you have any other comments?


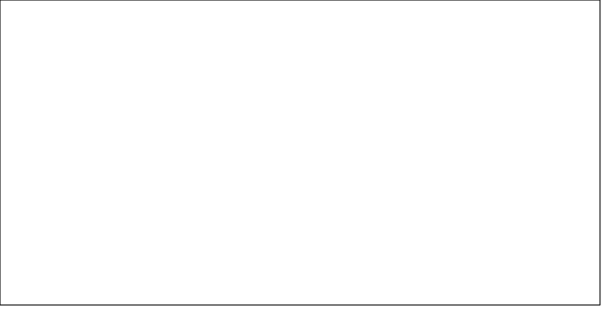


**Thank-you for completing this form.**

**Appendix 2: Recruitment adverts and request for personal information**

**Stakeholder Group for a review of physiotherapy for stroke**

**Project aim:** to bring together best evidence to do with physiotherapy for stroke

**Full description of involvement opportunity:** We are looking for up to 15 people to be in a Stakeholder Group to attend a series of meetings to steer some decisions about the best way to bring together, summarise and reach conclusions based on research evidence for physiotherapy for people who have had stroke.

We are keen to include a range of people with different experiences of physiotherapy for stroke. This includes stroke survivors, their family members and carers, and professionals involved in stroke rehabilitation delivery, education or service provision.

For further details, or to apply to be involved, please email Alex **by 31^st^ October**: [Alex.TodhunterBrown@gcu.ac.uk](mailto:Alex.TodhunterBrown@gcu.ac.uk)

**Request for personal details**

Dear XXXX

Thank you for your interest in joining the ‘Stakeholder Group’ for the SPRUCE project. This project is bringing together the most up to date evidence about how physical rehabilitation (physiotherapy) can help improve function, balance and walking after stroke. The attached Role Description gives some more information about the project and what members of the Stakeholder Group will do.

If you would like to be a member of the Stakeholder Group, please could you reply to this email with the answers to the ‘**questions about you’,** listed below, **by 8^th^ November**.

The Stakeholder Group can only have a maximum of 15 members, and we want these members to be as varied as possible. We will use your answers to these questions to help us select the people who will bring most variety to the Stakeholder Group. We will let you know by 12^th^ November whether or not we are able to offer you a place on the Stakeholder Group. Please note, that if you are invited to join the group, the first meeting will be held in the afternoon 25^th^ November.

Kind regards

Alex

**Questions about you**

To help us select people with different experiences to join our Stakeholder Group, please give brief answers to the questions that are relevant to you:

*If you are a stroke survivor or a family member or carer of a stroke survivor:*

1. What healthboard area / region do you live in?
2. How long ago was your stroke (or the stroke of your family member / person you care for?)
3. How does your stroke affect your/your family member’s walking / function / balance? (please select)

- Can walk independently (with no help or aids)
- Can walk with use of a walking aid and/or the supervision of another person
- Can walk with the help of another person
- Unable to walk (or very limited walking)

1. Why do you want to be part of this Stakeholder Group?
2. Can you attend all four meetings (see Role description for dates)?

*If you are a health professional who works with stroke survivors:*

1. What healthboard area / region do you work in?
2. How long have you worked with stroke survivors as a health professional?
3. What area of stroke care do you mainly work in (e.g. acute stroke unit, rehabilitation unit, community)?
4. Why do you want to be part of this Stakeholder Group?
5. Can you attend all four meetings (see Role description for dates)?

**Appendix 3: role description for physiotherapy professionals and stroke survivors**

**
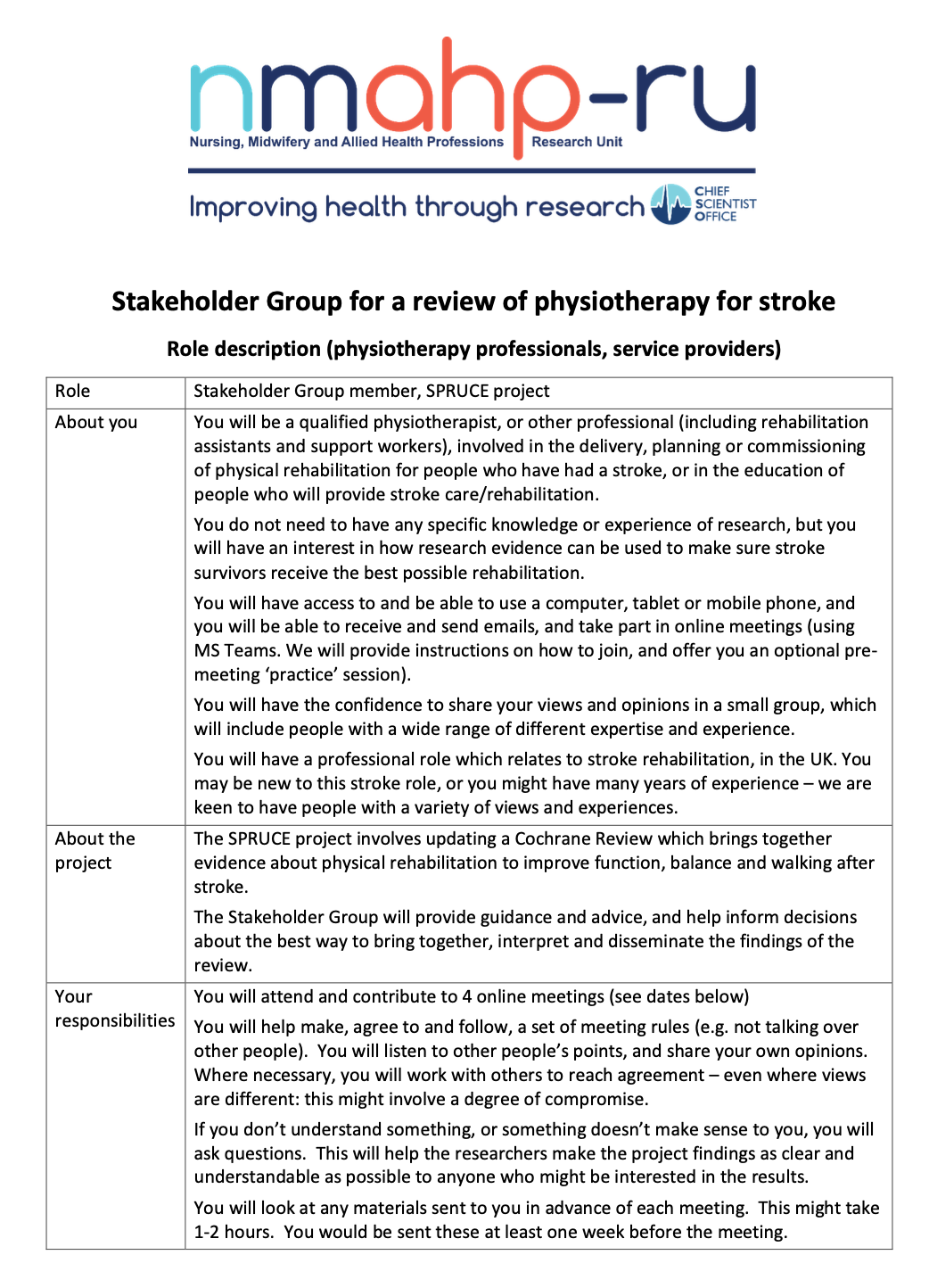
**

**
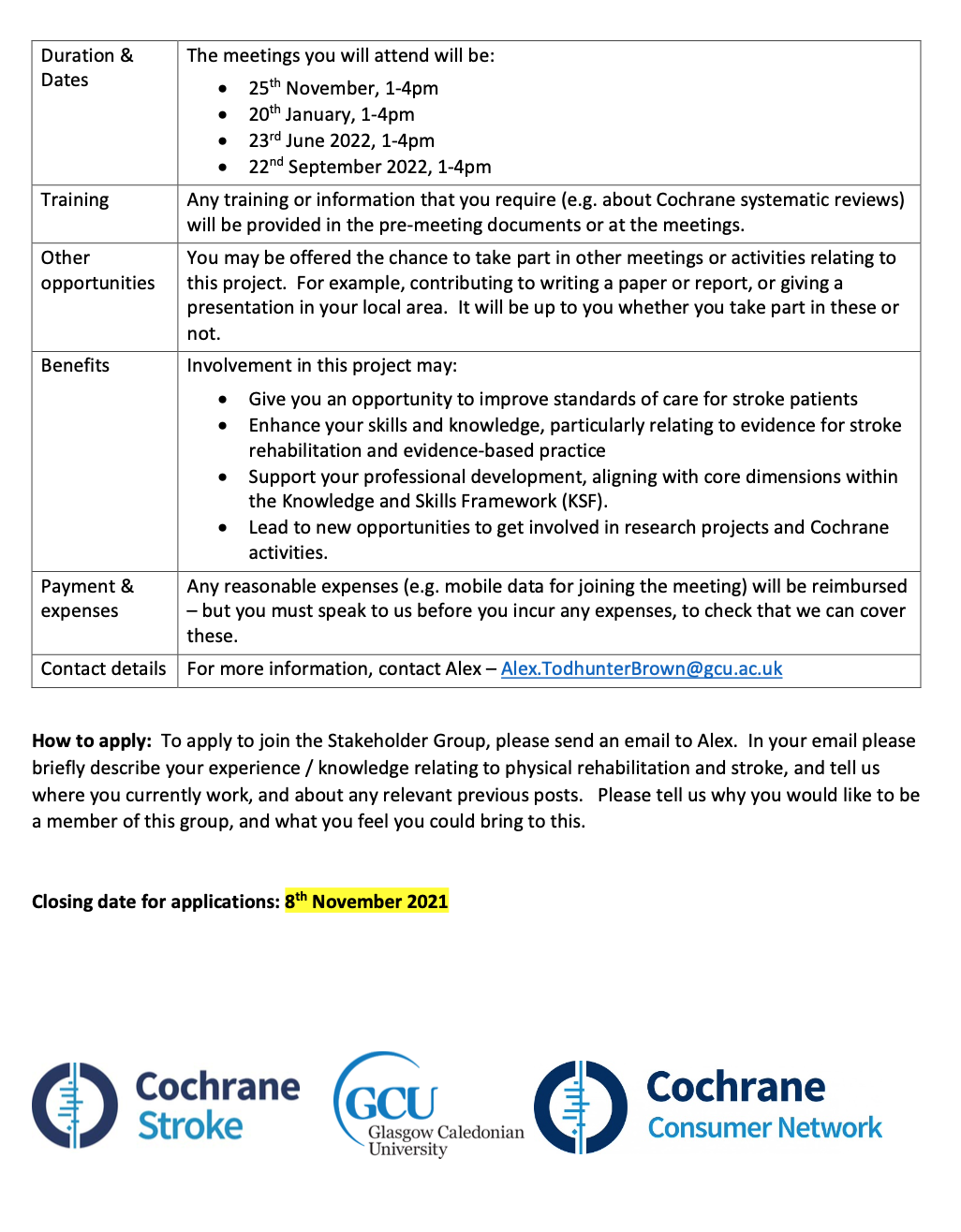
**


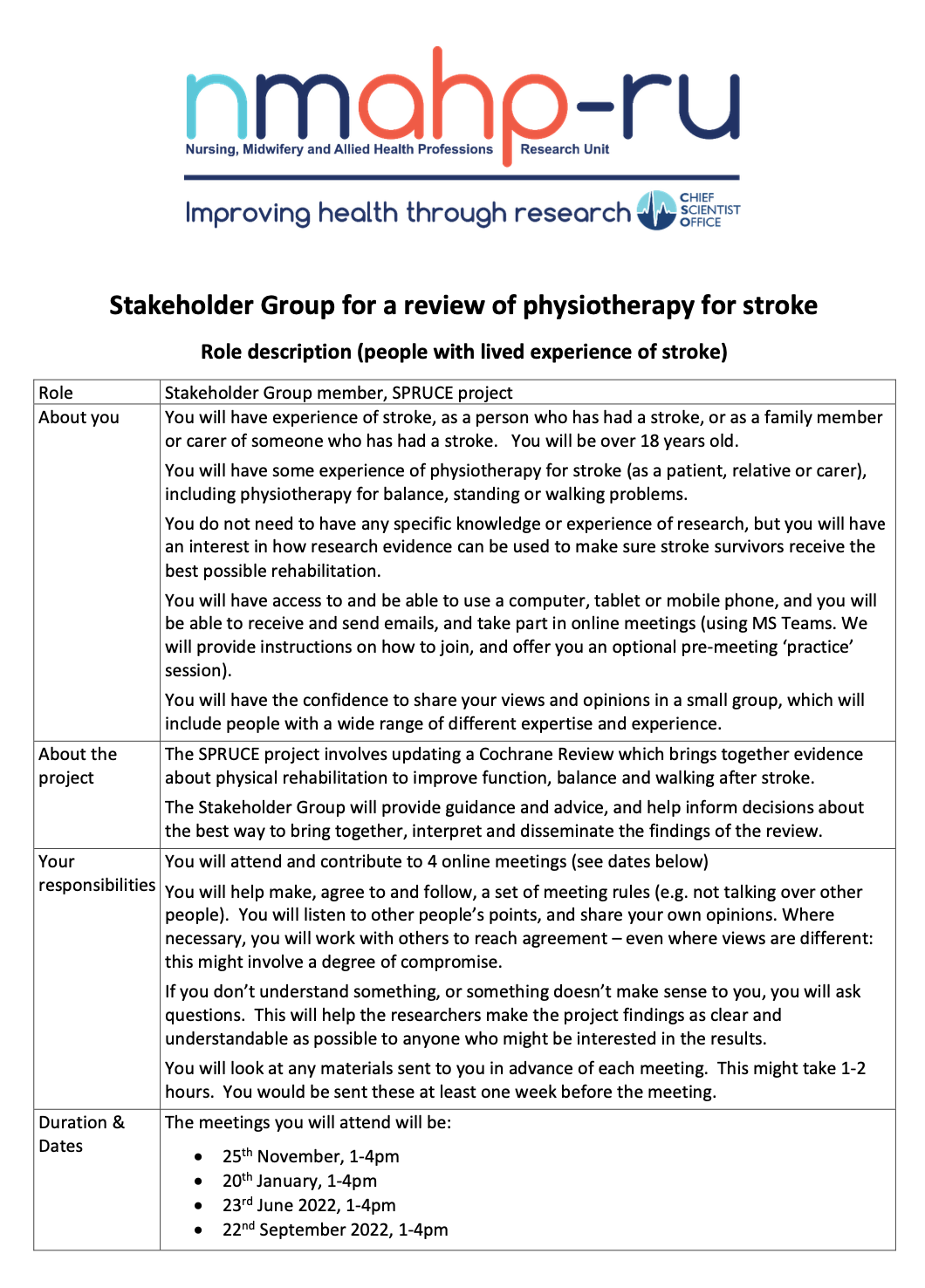


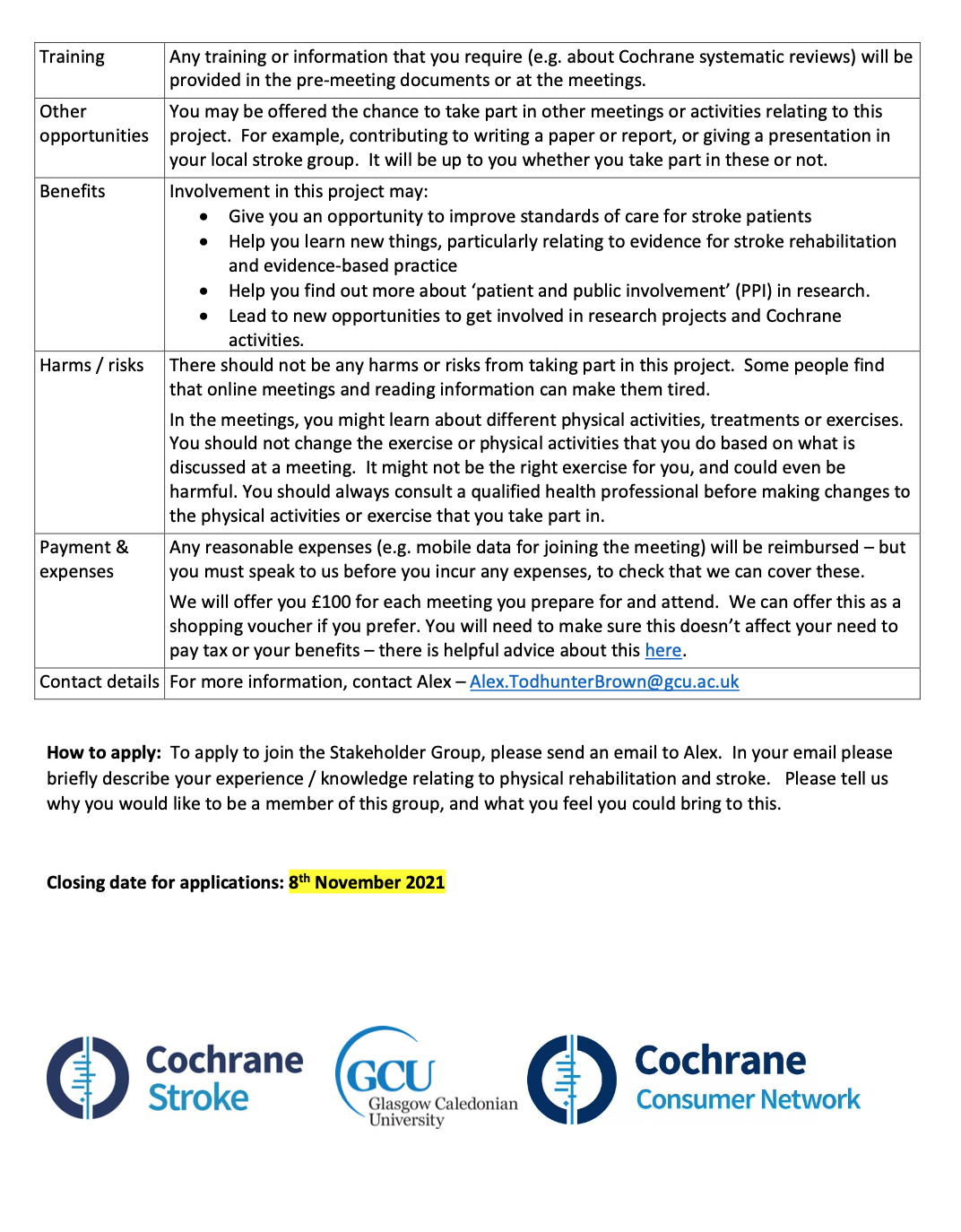


**Appendix 4: meeting rules**

**Agreed meeting rules**

- Consider sharing emails to check up on each other
- Please put camera on because easier to lip read
- Confidentiality – what’s said in the room stays in the room
- Let everyone have their say
- Please respect each other
- Allow people to be quiet during meeting
- ‘Parking’ area – acknowledge issues this cannot address but may be able to take forward to other forums
- Make sure someone is checking the chat box and can read out messages in chat box so that those without the chat function do not miss out
- Ask questions – put your hands up or message in chat if you need to clarify anything

**Appendix 5: Further details provided in response to the stakeholder reflections on the coproduction process**

1. ***Do you believe that your/stakeholders’ ideas were heard during the coproduction process? Prompt: why?***

Stakeholders considered that their ideas were heard by both the research team and the rest of the group; researchers agreed. Stakeholders commented that they felt their ideas were welcomed and discussed which often led to ideas being expanded upon or new ideas generated during discussion. Researchers commented on the need to include a variety of ways to gain engagement such as online meetings, updates and engagement via email as well as webinars and online voting. One researcher remarked that the online meetings made it difficult to know whether those who were quieter during meetings were happy with decisions and had nothing to add or had difficulty getting themselves heard in the online space despite regular check-ins during the meeting.

1. ***Do you believe organisers took your/stakeholders contributions to the coproduction process seriously and that your contributions influenced final decisions on the project? Prompt: why?***

All stakeholders considered that their contributions were taken seriously and influenced the project. Stakeholders commented on the benefit of research team taking notes during the meeting and summarising key points at the end of the session. One stakeholder identified the benefit of the virtual environment for allowing everyone to view the live notes on a ‘shared slide’ to see how discussions progressed. Researchers highlighted the importance of involving stakeholders in a meaningful way, demonstrating that ideas had been heard, contributions valued and taken seriously and demonstrated the impact that stakeholders’ ideas made throughout each meeting then recapping at subsequent meetings. One researcher commented that the lead researcher – and meeting chair – modelled an open approach to stakeholder involvement and was confident to say ‘I hadn’t thought of that’ or ‘that was a good point’ then following up with on ideas and discussions. The coproduced framework to explain physical rehabilitation after stroke was a good example of the research team starting with one idea, and completely changing it based on the stakeholder input.

1. ***Did you feel you/stakeholders were able to clearly express your viewpoints and all participants were given equal opportunity to participate in discussions? Prompt: why?***

Stakeholders considered that they were mostly able to express their views, but one stakeholder suggested the use of breakout rooms may have been more beneficial to facilitate discussion within a smaller group before feeding back to the larger group. The online environment was considered largely positive, predominantly due to the ability to contribute without the travel time or juggling other commitments. However, stakeholders also acknowledged the online platform sometimes stilted the experience of sharing, and technology could take a bit of time to get used to or need to be updated to ensure it ran smoothly. Researchers highlighted, like the stakeholders, that the online environment made it difficult to ensure that everyone could participate equally. The group developed ‘ground rules’ co-created at the start of the first meeting went some way to ensure a respectful environment. One researcher reflected on the fact that some stakeholders chose to leave the group because they felt unable to contribute; the researcher felt that this may have been due to the complex nature of the information discussed. While researchers made efforts to make information as accessible as possible in subsequent sessions, and provide different ways to feedback (e.g. ‘chat’ text box function during meeting, Microsoft teams page after meetings, via email, anonymous voting), they still expressed concerns that they were asking stakeholders to contribute to complex – often theoretical – debates, which may have been difficult for all participants to contribute to. Some discussions becoming very ‘professional’ and ‘complex’ and suggestions to stop and provide more focussed attention to carers and stroke survivors, then these discussions might have been better in a break out room, giving others more ‘space’ to speak their own lived experience and viewpoints.

1. ***To what extent was information made available to you/stakeholders either before or during the engagement process to help you participate knowledgeably in the process?*** **Prompt: what was good about the information given? What could have been better? Was information available in a format or language that was suitable for you?**

Stakeholders and researchers considered that information was shared well prior to, during and after each meeting. Stakeholders reflected on the benefit of providing feedback in a variety of forms, plus commented that the pre-recorded videos shared before meetings were helpful at providing time to digest complex information, and as a supplement for those who couldn’t attend meetings due to diary clashes. One stakeholder commented that the information was sometimes complex and hard to understand. One researcher highlighted that it was sometimes challenging providing information before meetings due to the fast paced nature of the review process. Before meeting 5 researchers circulated a pre-recorded version of the slides and material for the meeting. Stakeholders reflected that having the pre-recorded presentation that they were really useful, providing time to think, listen, repeat information, reflect, form questions, and focus the mind on the aim of the meeting. This pre-meeting recording was helpful to prepare and be ready to use time in meeting to full effect.

1. ***What could we have done differently to make you/stakeholders feel that you were valued, important and heard? Prompt: why?***

Stakeholders did not respond specifically to this question, instead they suggested things that could be improved under different questions (e.g. shorter meetings, more breaks, breakout rooms, simplify complex information more etc.). Researchers highlighted that, despite their best efforts to make sure contributors were valued, researchers could have planned for more time to support the stakeholders. The researchers also highlighted that they felt it would be important before any similar stakeholder events to identify a suitable software option for voting online during meetings, as this can make it easier for stakeholders views to be captured. A mix of online and in person meetings may have been a better way to develop cohesiveness/rapport, understanding and participation in discussion.

1. ***If relevant, was your/stakeholders ethnicity or culture considered? Prompt: why?***

Stakeholders did not respond to this question. Researchers highlighted that they did not consider ethnicity or culture when recruiting stakeholders. In future, criteria for diversity could be improved by expanding the criteria especially in relation to black and ethnic minorities.

1. ***Do you feel there was a wide enough group of stakeholders from all backgrounds with good representation and diversity? Prompt: why?***

One stakeholder answered this question and felt that there was a wide enough representation of diverse backgrounds with a rich mix of professionals, stroke survivors and carers. Researchers highlighted that this is an area that they could have looked at differently, particularly in relation to black and ethnic minorities. Researchers did not ask about deeper aspects of for example, class, education, ethnicity, when recruiting to this stakeholder group. Furthermore, researchers did not capture information about potential conflicts of interest. The recruitment criteria focused on “purposefully” recruiting stakeholders who (i) lived in different parts of the UK, (ii) were varied in time post stroke, and (iii) had different levels of impairment / disability (or in the case of the physios: (ii) varied years of experience as a physio, (iii) worked in different clinical settings). Researchers commented that adding in additional criteria may have made the recruitment process more challenging (e.g. due to general data protection regulations and the handling of personal data), but on reflection, this is something that would warrant further consideration in future reviews. There was a diverse stakeholder group when considering the initial criteria however this could be improved in future by expanding the recruitment criteria. In relation to potential conflicts of interest among the final stakeholder group, researchers reflected that this work would have been strengthened by having a clear strategy for the management of conflicts of interest.
